# Expression analysis following argon treatment in an *in vivo* model of transient middle cerebral artery occlusion in rats

**DOI:** 10.1186/2045-9912-4-11

**Published:** 2014-06-06

**Authors:** Astrid V Fahlenkamp, Mark Coburn, Antonio de Prada, Nadine Gereitzig, Cordian Beyer, Hajo Haase, Rolf Rossaint, Jens Gempt, Yu-Mi Ryang

**Affiliations:** 1 Department of Anesthesiology, University Hospital RWTH, Pauwelsstr. 30, Aachen 52074, Germany; 2 Department of Neurosurgery, Klinikum rechts der Isar, Technische Universität, München, Germany; 3 Institute of Neuroanatomy, Faculty of Medicine, RWTH, Aachen, Germany; 4 Institute of Immunology, University Hospital RWTH, Aachen, Germany

**Keywords:** Argon, Noble gas, MCAO, Ischemia, Neuroprotection

## Abstract

**Background:**

Argon treatment following experimental neurotrauma has been found neuroprotective in an array of *in vivo* and *in vitro* models. The inherent cellular and molecular mechanisms are still unknown. We seeked to shed light on these processes by examinig the cellular distribution and the expression of inflammatory markers and growth factors in argon treated brain tissue.

**Methods:**

Male adult Sprague-Dawley rats were randomly assigned to one of the study groups: sham surgery + placebo, sham surgery + argon, tMCAO + placebo, and tMCAO + argon. Animals underwent 2 h-transient middle cerebral artery occlusion (tMCAO) using the endoluminal thread model or sham surgery without tMCAO. After the first hour of tMCAO or sham surgery a 1 h inhalative argon (50% argon/50% O_2_) or placebo (50% N_2_/50% O_2_) treatment was performed. Brains were removed and evaluated after 24 h. RealTime-PCR was performed from biopsies of the penumbra and contralateral corresponding regions. Paraffin sections were immunostained with antibodies against GFAP, NeuN, and Iba1. Cell counts of astrocytes, neurons and microglia in different cortical regions were performed in a double-blinded manner.

**Results:**

Fifteen animals per tMCAO group and twelve sham + placebo respectively eleven sham + argon animals completed the interventional procedure. We identified several genes (IL-1β, IL-6, iNOS, TGF-β, and NGF) whose transcription was elevated 24 h after the study intervention, and whose expression levels significantly differed between argon treatment and placebo following tMCAO. Except for the core region of ischemia, cell numbers were comparable between different treatment groups.

**Conclusion:**

In our study, we found an elevated expression of several inflammatory markers and growth factors following tMCAO + argon compared to tMCAO + placebo. Although conflicting the previously described neuroprotective effects of argon following experimental ischemia, these findings might still be associated with each other. Further studies will have to evaluate their relevance and potential relationship.

## Introduction

Cerebral ischemia and its sequelae is one of the leading causes of death and long-term disability worldwide [1,2]. In contrast to its common incidence, therapeutic options are limited. Due to the high vulnerability of neuronal tissue to oxygen deprivation, recanalization procedures have to take place in a limited time slot of 3 h and up to 4.5 h in special cases [1]. The area of greatest interest is the so called penumbra: the transition zone in between ischemic core and healthy tissue, where neurons are in a state of hibernation that can be restituted ad integrum with optimum treatment [3]. Numerous experimental therapeutic approaches have focused on protection and salvation of neurons in this area. While most of these therapeutics showed promising results in experimental studies, none to negative effects were seen in clinical trials [1]. Until today, and with limited recommendation, only therapeutic hypothermia has found its way into cerebral ischemia treatment guidelines [1,2].

The noble gas argon has recently come into focus as a potential adjunctive neuroprotective agent: protective effects of noble gases have been widely shown in different experimental models [4-6], and argon seems to have similar beneficial effects on injured tissue [7-10]. Moreover, unlike xenon, it lacks sedative side effects under normobaric conditions [11], and is cheaper due to its higher fraction in normal atmosphere. These features plus expectedly little disadvantageous side effects in humans make argon a promising candidate. However, while neuroprotective effects of argon have already been demonstrated, data on its cellular actions are limited: *In vitro*, argon activated a cellular enzyme, and it only slightly interfered with the inflammatory microglial response following stimulation with bacterial lipopolysaccharide [12]. *In vivo,* an elevated expression of an anti-apoptotic protein was found elevated following argon treatment in neonatal asphyxia [7]. Argon’s protective action was not exerted via NMDA-antagonism by interaction with glycine in a model of traumatic brain injury [9]. The mechanisms for argon’s neuroprotective actions in cerebral ischemia are unknown.

The aim of this study was to shed first light on argon’s mechanisms of action in a stroke model. 24 h after transient middle cerebral artery occlusion (tMCAO) [13] and a delayed additional administration of 50% argon as described previously [10], we performed a gene expression analysis of inflammatory and growth factors and examined the distribution of vital neurons, microglia and astrocytes in the penumbra.

## Methods

### Study design and animal enrolment

This experimental study was in part designed as a subgroup- and follow-up analysis of the above mentioned efficacy analysis of argon in MCAO [10], with additional enrolment of animals obtaining the same experimental procedures. Animal research and care procedures were approved by the governmental review board (Landesamt für Natur, Umwelt und Verbraucherschutz, Germany) beforehand. Species-appropriate housing was assured in macrolone cages in a specified pathogen-free environment, with food and water *ad libitum* and a 12 h-light-dark-cycle.

### Transient middle cerebral artery occlusion (tMCAO)

Male Sprague Dawley rats (250-295 g, Harlan Laboratories, Netherlands) were randomly assigned to one of the following groups: a) sham surgery + placebo (Sham N_2_): n = 12; b) sham surgery + argon treatment (Sham Ar): n = 12; c) tMCAO + placebo (tMCAO N_2_): n = 15; and d) tMCAO + argon (tMCAO Ar): n = 15. One animal in the Sham Ar group was lost before completion of the 24 h follow-up due to other than intervention-associated reasons.

**Figure 1:**
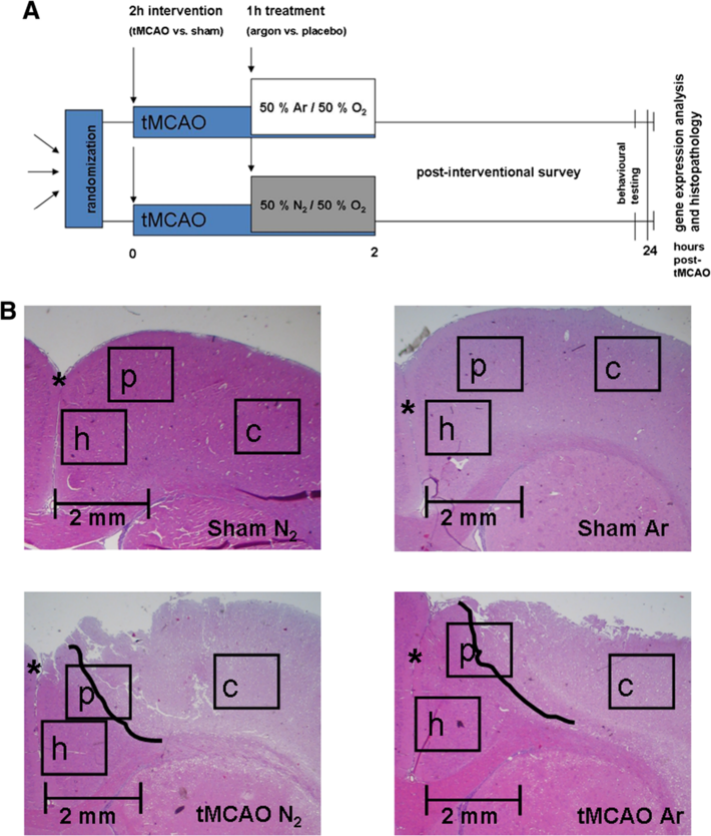
**Study procedure and exemplary histological graphics.** A simplified graphic of the study procedure is shown in **A**. After randomization animals received i.p. anesthesia and tMCAO or sham surgery. 1 h after the occlusion of the MCA with a thread treatment with 50% argon/50% oxygen or placebo (a 50% nitrogen/oxygen-mixture) was performed by mask inhalation for another hour. After that, treatment and MCA occlusion were terminated. Animals were left to recover from the procedure. 24 h later after behavioral testing, animals were sacrificed and brains obtained for further experiments. Example pictures of the HE-stained penumbra regions (slice 3) from the tMCAO + argon (MCAO Ar) and tMCAO + placebo (MCAO N_2_) groups vs. corresponding regions from sham + argon (Sham Ar) and sham + placebo (Sham N_2_) are shown in **B**. The demarcation zone between penumbra and necrotic tissue is tagged with a bold black line. The interhemispheric fissure is asterisked. The positions of the images taken for cell evaluation and quantification are marked by black rectangles (h healthy cortex; p penumbra; c ischemic core).

The interventional procedure is pictured in Figure 1A. Following induction of anaesthesia (0,15 mg/kg medetomidine, 2 mg/kg midazolam and 0.005 mg/kg fentanyl i.p.), animals underwent 2 h of tMCAO with the intraluminal thread-occlusion technique [10,13] or sham surgery (identical surgical procedure without intraluminal thread-occlusion). Full occlusion of the middle cerebral artery (MCA) was assured by laser Doppler flowmetric assessment of the blood flow in this vessel (PeriFlux System 5000, Type PF 5001, Perimed, Sweden) as previously described [10]. Throughout the surgical procedure, animal body temperature was sustained at 37°C. Anaesthesia was maintained by repeated i.p. doses of 0.1-0.15 ml of the above described anesthetic mixture in 1 h intervals, if applicable titrated to clinical needs. Heart rate, arterial oxygen saturation and mean arterial blood pressure were continuously assessed, blood gases and pH were repeatedly measured to verify sufficient spontaneous breathing and circulation. At the end of the first hour after induction of tMCAO (or sham surgery), animals were treated according to their randomization with either placebo (nitrogen) or argon for another hour until reperfusion [10]. Argon or placebo were applied in an inspiratory concentration of 50% mixed with 50% oxygen via facial mask and spontaneous ventilation of the i.p. anesthetized animals. Sufficient ventilation was guaranteed by regular blood gas analyses for normoxia and normocarbia [10]. After 2 h of tMCAO including 1 h of treatment, reperfusion was restored by removal of the thread in the tMCAO animals. Following surgical closure of skin wounds and preemptive pain therapy (metamizole 15-50 mg/kg body weight), all animals were returned to their cages for recovery. Animals underwent neurological and behavioral testing [10,14] directly before sacrifice at 24 h *post interventionem.*

### Tissue preparation for gene expression analysis

Rat brains were removed immediately after sacrifice in deep anesthesia, cut into seven 2 mm thick coronal sections using a rat brain matrix (Plastics One Inc., USA) and stained at 37°C for 15 min in a 2% solution of 2,3,5-triphenyltetrazolium chloride (TTC) in normal saline to discriminate vital from dead tissue [15,16]. Images of the TTC-stained sections were acquired (Sony DXC 390P, Sony Germany) before cryo-preservation at -80°C until further processing. Infarct volumes were assessed with an image analysis software (Optimas 6.5; Adept Electronic Solutions Pty Ltd, Sydney, Australia). Addition of all cross-sectional infarcted coronal brain sections which were multiplied by 2 (2 mm slice thickness) rendered the total infarct volumes of each infarcted brain [10]. Infarct volumes were edema-corrected by use of this formula [17]:

$$\mathrm{Vedi}=\mathrm{Vinfarct}*\left(1-\left(\mathrm{Vipsi}-\mathrm{Vcontra}\right)/\mathrm{Vcontra}\right)$$

V edi = Volume edema-corrected infarct volume, V infarct = Volume infarct, V ipsi = Volume ipsilateral infarcted hemisphere, V contra = Volume contralateral non-infarcted hemisphere.

### RNA isolation

On the basis of TTC-staining, the penumbra regions of the infarcted brain hemispheres were identified. Total RNA was isolated from the cortical penumbra of the infarcted hemisphere and corresponding contralateral regions from three consecutive coronal sections of each brain (sections 3, 4 and 5) employing NucleoSpin RNA II (MACHEREY-NAGEL, Germany) according to the manufacturers’ instructions. Briefly, brain tissue was homogenized (5000 g, 15 s) with Precellys Keramik-Kit (PEQLAB Biotechnologie, Germany). Cells were further lysated with β-mercaptoethanol, filtrated by centrifugation and washed in 70% ethanol. DNA was digested and RNA purified in several washing steps and eluted in ultra-purified water (Invitrogen, Germany).

### Gene expression analysis

RNA concentrations and purity were measured using the NanoDrop1000 (PEQLAB Biotechnologie, Germany). Reverse transcription reactions were performed with M-MLV RT-kit (Invitrogen, Germany) according to the manufacturer’s instructions and random hexanucleotide primers (Invitrogen, Germany). Real-time rtPCR (RT-rtPCR) reactions were carried out in a mixture containing 2 μl cDNA, 2 μl RNAse-free water (Invitrogen, Germany), 5 μl 2 × Sensi Mix × Plus SYBR & Fluorescein Kit (Quantace, Bioline, Germany) and 0.5 μl primers (10pmol/μl). Primer sequences are listed in Table 1. Reactions were conducted in standard tubes using the MyiQ RTrtPCR detection system (Bio-Rad Laboratories, Germany) under the following conditions: 10 min enzyme activation at 95°C, 40 times 15 s denaturation at 95°C then 30 s annealing at individual temperatures then 30 s amplification at 72°C, and 5 s fluorescence measurement at 72°C. Internal standard curves to assess the concentration of the target gene in the samples were generated by measuring several dilutions of a target gene pool with each reaction. Melting curve analysis and agarose gel electrophoretic evaluation of the RT-rtPCR products were routinely performed to determine the specificity of the RT-rtPCR reaction. Gene expressions were normalized to the mean expression values of the housekeeping gene cyclophilin A; the expression levels of the infarcted hemispheres were furthermore set in relation to their corresponding contralateral hemispheres.

**Table 1:** Primer sequence, product length and characteristics

**Transcript**	**Sequence**	**Orientation**	**Annealing temperature (°C)**	**Transcript size (base pairs)**	**Melting point (°C)**	**GeneBank_ID**
Cyclophilin	GGCAAATGCTGGACCAAACAC	Sense	60	196	82	NM_017101.1
	TTAGAGTTGTCCACAGTCGGAGATG	Antisense				
Tumor nekrose faktor alpha	CTCCCAGAAAAGCAAGCAACC	Sense	57	210	85	NM_012675.3
(TNFα)	CGAGCAGGAATGAGAAGAGG	Antisense				
Interleukin-1β	CTGTGACTCGTGGGATGATG	Sense	60	210	81	NM_031512.2
(IL-1β)	GGGATTTTGTCGTTGCTTGT	Antisense				
Interleukin-6	CCGGAGAGGAGACCTCACAG	Sense	60	161	78	NM_012589.1
(IL-6)	ACAGTGCATCATCGCTGTTC	Antisense				
Chemokine (C-C) ligand 2	CCAGAAACCAGCCAACTCTC	Sense	59	192	83	NM_031530.1
CCL2	CCGACTCATTGGGATCATCT	Antisense				
Matrix metalloproteinase-9	CCACCGAGCTATCCACTCAT	Sense	60	180	79	NM_031055.1
MMP-9	GTCCGGTTTCAGCATGTTTT	Antisense				
Hypoxia inducible factor 1	TCAAGTCAGCAACGTGGAAG	Sense	60	198	81	NM_024359.1
alpha (HIF-1α)	TATCGAGGCTGTGTCGACTG	Antisense				
Chemokine (C-C) ligand 5	TGCCCACGTGAAGGAGTATTTTTA	Sense	60	81	78	NM_031116.3
(CCL5)	TGGCGGTTCCTTCGAGTGACAA	Antisense				
Transforming growth factor β (TGF-β)	GGGACTCTCCACCTGCAAGAC	Sense	63	393	88	NM_012578.2
	CTCTGCAGGCGCAGCTCTG	Antisense				
Nerve growth factor	GAAACGGAGACTCCGTTCACC	Sense	60	100	82	NM_001067130.1
(NGF)	GATTGTACCATGGGCCTGGA	Antisense				
Vascular endothelial growth factor α (VEGFα)	CCTGGTGGACATCTTCCAGGAGTACC	Sense	60	196	84	NM_031836.2
	GAAGCTCATCTCTCCTATGTGCT	Antisense				
Inducible NO-synthetase	CACCTTGGAGTTCACCCAGT	Sense	59	179	85	NM_012611.3
(iNOS)	ACCACTCGTACTTGGGATGC	Antisense				
CD3	AGAACTGCATGGAGGTGGAC	Sense	60	232	84	NM_001108140.1
	TTTCGGATGGGCTCATAGTC	Antisense				
CD11b	TTACCGGACTGTGTGGACAA	Sense	62	219	82	NM_012711.1
	AGTCTCCCACCACCAAAGTG	Antisense				
2’3’-Cyclic nucleotide 3’ phosphodiesterase (CNP)	TGGCGAAGAAGATGGAAGTCA	Sense	60	257	82	M18630.1
	GTGGGTGAAGGAACTGATGGTT	Antisense				
Glial fibrillary acid protein	AGAAAACCGCATCACCATT	Sense	60	188	83	NM_017009.2
(GFAP)	GCACACCTCACATCACATCC	Antisense				
Insulin-like growth factor 1	TGATCTGAGGAGGCTGGAGATGTA	Sense	59	95	82	NM_178866.4
IGF-1	CTTCTGAGTCTTGGGCATGTCA	Antisense				

A full summary of the primer sequences employed for gene expression analysis plus their characteristics is given in Table 1.

The primer sequences and further information about the transcripts we analyzed is given in Table 1. We selected the transcripts for analysis as follows: Argon was found to have effects on both glial and neuronal cells, and it only slightly attenuated LPS-induced production of cytokines IL-1β, TNFα and IL-6 in a microgial cell line [12]. HIF-1α was found to be induced in xenon but not in argon renal cell protection [18]. Since knowledge about argon effects is in general very limited, additional factors that are released by neuronal or glial or invading immune cells and are furthermore associated with damage and/or repair following neurotrauma were chosen for analysis.

### Immunhistochemistry (IHC)

In deep anesthesia, rats were transcardially perfused with 2% (w/v) paraformaldehyde (Roth, Germany) containing 15% (v/v) saturated picric acid at pH 7.4 (AppliedChem, Germany). Brains were removed, kept in the same fixative overnight and paraffin-embedded the next day (Merck, Germany). Coronary sections (slices 3 and 5) of 5 μm thickness were prepared, mounted on glass slides and rehydrated using standard protocols. To verify infarction (MCAO) or integrity (Sham), sections were stained with hematoxylin/eosin (HE) using a standard protocol (Exemplary pictures are presented in Figure 1B). For IHC, sections were incubated with blocking serum for 1 h at room temperature, following exposure to primary antibodies overnight (anti-NeuN (1:500, Abcam, Cambridge, UK), anti-GFAP (1:1000, Abcam, Cambridge, UK), and anti-ionized calcium-binding adaptor molecule 1 (Iba1, 1:250, Wako, Osaka, Japan). After several washing steps, sections were incubated with biotin-conjugated secondary antibody for 1 h, and subsequently with a biotin–avidin–enzyme complex (Vectastain ABC kit, Vector Laboratories, Burlingame, USA). Antibody binding was visualized by substrate incubation (AEC Substrate kit, Invitrogen, Camarillo, USA). To quantify vital cells, standard light microscopy with a 20× objective lens was performed and images were taken of three defined regions (healthy cortex, cortex penumbra and ischemic core, see Figure 1B) in the infarcted hemisphere and of corresponding contralateral regions with a standard digital camera. Vital neurons (NeuN positive cells with a vital large light caryon without shrinked/dense apperance), astrocytes (GFAP-positive cells with minimum three branches) and microglia (Iba1-positive cells with minimum two rami) were analyzed with the help of a cell count software (ImageJ, http://rsbweb.nih.gov/ij/) by two independent examiners blinded to the treatment.

### Statistics

Parametric data were evaluated by oneway ANOVA, followed by post-hoc Newman-Keuls-testing for statistical significance. GraphPad PRISM (GraphPad Software Inc., La Jolla, California, USA) was used to calculate the statistics and generate the figures. All values are given as means ± SEM. A p-value ≤ 0.05 was considered statistically significant.

## Results

### Expression of inflammatory genes

**Figure 2:**
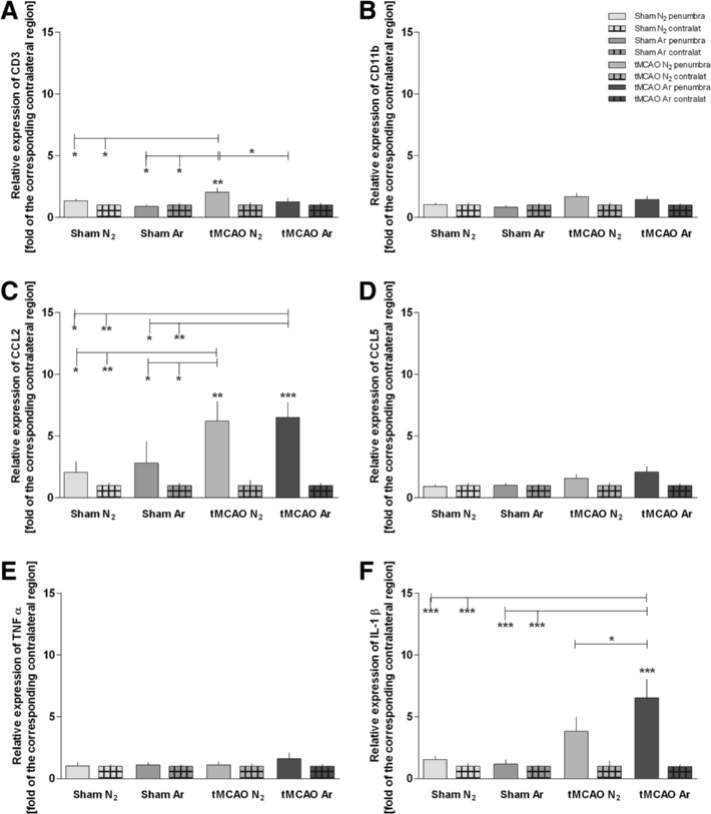
**Expression levels of inflammatory genes.** Expression levels of different inflammatory genes measured by RT-PCR are shown in Figure 2 (**A**: CD3; **B**: CD 11b; **C**: CCL2; **D**: CCL5; **E**: TNFα; **F**: IL-1β). Samples were taken from the penumbra in tMCAO or corresponding region in sham animals plus corresponding contralateral regions (Sham N_2_ n = 9; Sham Ar n = 8; tMCAO N_2_ n = 12; tMCAO n = 12). All values are presented as mean ± SEM. *p < 0.05; **p < 0.01; ***p < 0.001.

**Figure 3:**
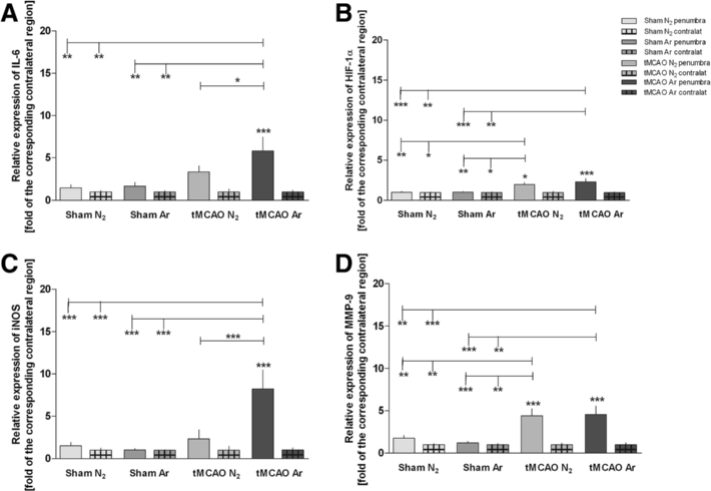
**Expression levels of further factors involved in post-ischemic inflammation and repair.** Expression levels of different factors involved in post-ischemic inflammation and repair processes measured by RT-PCR are shown in Figure 3 (**A**: IL-6; **B**: HIF-1α; **C**: iNOS; **D**: MMP9). Samples were taken from the penumbra in tMCAO or corresponding region in sham animals plus corresponding contralateral regions (Sham N_2_ n = 9; Sham Ar n = 8; tMCAO N_2_ n = 12; tMCAO n = 12). All values are presented as mean ± SEM. *p < 0.05; **p < 0.01; ***p < 0.001.

Expression of the cell marker protein CD3 was significantly higher in the penumbra of tMCAO + placebo animals compared to tMCAO + argon or sham (Figure 2A). Chemokine CCL2-expression levels were significantly elevated in both tMCAO (+argon/+placebo) groups in comparison to sham, with no significant difference between tMCAO groups (Figure 2C). Interleukines’ IL-1β and IL-6 expression was significantly elevated in the penumbra of the tMCAO + argon group compared to tMCAO + placebo (IL-1β: 1.7 fold increase of induction, p < 0.05; IL-6: 1.7 fold increase of induction, p < 0.05; Figure 2F, 3A). IL-1β and IL-6 expression was significantly elevated in tMCAO + argon but not in tMCAO + placebo penumbra compared to the sham animals (Figure 2F, 3A). The same was found for iNOS expression, which was significantly induced in the penumbra of the tMCAO + argon group (3.5 fold increase of induction compared to tMCAO + placebo, p < 0.001), but not in the penumbra of the tMCAO + placebo group (Figure 3C) compared to the sham animals. CD11b, CCL5 and TNFα expression levels were not significantly elevated at 24 h following cerebral infarction compared to healthy controls (Figures 2, B, D, E). The expression of matrix metalloproteinase-9 (MMP-9) and hypoxia inducible factor (HIF)-1α were significantly higher in the penumbra following cerebral ischemia compared to healthy controls, with no significant difference between tMCAO + argon and tMCAO + placebo (Figures 3B, D).

### Expression of growth factors

**Figure 4:**
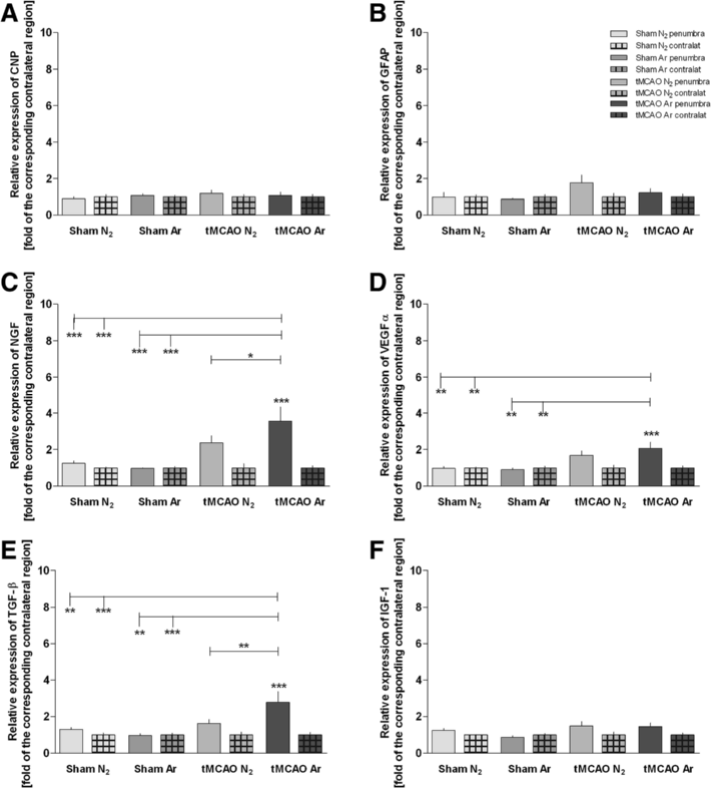
**Expression levels of repair and growth factors.** Expression levels of different growth factor genes involved in cerebral cell differentiation and measured by RT-PCR are shown in Figure 4 (**A**: CNP; **B**: GFAP; **C**: NGFb; **D**: VEGF; **E**: TGF β; **F**: IGF-1). Samples were taken from the penumbra in tMCAO or corresponding region in sham animals plus corresponding contralateral regions (Sham N_2_ n = 9; Sham Ar n = 8; tMCAO N_2_ n = 12; tMCAO n = 12). All values are presented as mean ± SEM. *p < 0.05; **p < 0.01; ***p < 0.001.

The expression levels of 2',3'-cyclic-nucleotide 3'-phosphodiesterase (CNP), glial fibrillary acid protein (GFAP), and insulin-like growth factor (IGF)-1 were not affected 24 h after the intervention in the different study groups (Figures 4A, B, F). A significant upregulation of transforming growth factor (TGF)-β, and neuronal growth factor (NGF) mRNA-levels was found in the penumbra 24 h following tMCAO + argon treatment compared with tMCAO + placebo (TGF-β: 1.7 fold increase of induction, p < 0.01; NGF: 1.5 fold increase of induction, p < 0.05; Figures 4C, E). TGF-β and NGF expression was found significantly elevated in tMCAO + argon but not in tMCAO + placebo penumbra when compared to sham (Figures 4C, E). Argon treatment following tMCAO further led to a significantly increased expression of vascular endothelial growth factor (VEGF)α in the penumbra compared to sham, but not to tMCAO + placebo (Figure 4D).

### Distribution of neurons, microglia, and astrocytes

#### Neu-N staining

**Figure 5:**
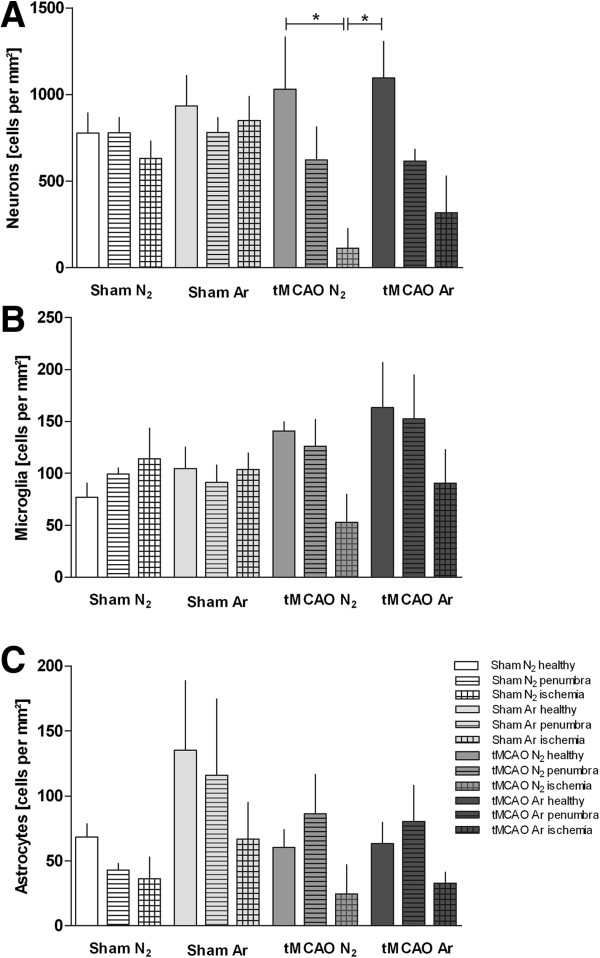
**Cell numbers of neurons, astrocytes and microglia in healthy cortex, penumbra and ischemic core region.** The numbers of neurons **(A)**, microglia **(B)** and astrocytes **(C)** differentiated by immunohistochemical staining in healthy ipsilateral cortex (healthy), penumbra (penumbra) and ischemic core (ischemia) of tMCAO animals and their corresponding sham regions are shown in Figure 5. (Sham N_2_ n = 3; Sham Ar n = 3; tMCAO N_2_ n = 3; tMCAO n = 3). All values are presented as mean ± SEM. * p < 0.05.

**Figure 6:**
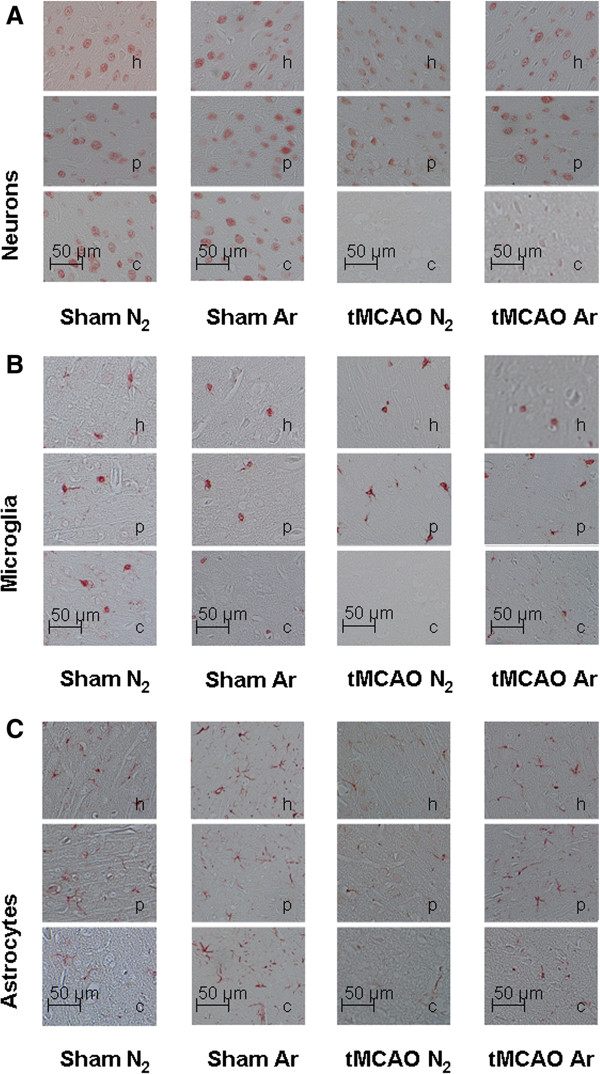
**Immunhistochemical stainings of neurons, astrocytes and microglia in healthy cortex, penumbra and ischemic core region.** Example pictures of neurons (NeuN staining, **A)**, microglia (Iba-1 staining, **B)** and astrocytes (GFAP staining **C)** taken from healthy ipsilateral cortex (h), penumbra (p) and ischemic core (c) of tMCAO animals and their corresponding sham regions are shown in Figure 6. (Sham N_2_ n = 3; Sham Ar n = 3; tMCAO N_2_ n = 3; tMCAO n = 3).

24 h following tMCAO versus sham surgery, numbers of vital neurons in the healthy contralateral hemispheres were comparable in all study groups (data not shown). In the ischemic core of the infarcted hemispheres, vital neurons were significantly reduced following tMCAO + placebo (Figure 5A) when compared to healthy ipsilateral regions or healthy tMCAO + argon. No significant differences were found between tMCAO + argon healthy and ischemic tissue, or between tMCAO + placebo and tMCAO + argon penumbra or ischemic core (Figure 5A). Example stainings are shown in Figure 6A.

#### Iba-1 staining

Microglia counts were comparable in healthy contralateral hemispheres in all study groups (data not shown). Microglia numbers were not significantly different among treatment groups neither in the ischemic nor in the penumbra or healthy region (Figure 5B). Example stainings are shown in Figure 6B.

#### GFAP-staining

Astrocyte numbers were not significantly different among treatment groups neither in the ischemic (Figure 5C) nor in the contralateral hemispheres (data not shown). Example stainings are shown in Figure 6C.

## Discussion

Recent experimental studies were able to demonstrate neuroprotective properties of argon in different ischemic and traumatic settings [6-10,19]. However, cellular and molecular processes leading to this neuroprotection are largely unknown. With this study, we intended to start elucidating protective mechanisms of argon treatment in a model of transient focal cerebral ischemia in rats. We identified several genes whose transcription was elevated 24 h after intervention, and whose expression levels significantly differed between argon treatment and placebo following tMCAO. Apart from the ischemic core in tMCAO + placebo animals, numbers of astrocytes, microglia and neurons were not significantly different in the study groups.

Following tMCAO + argon, several inflammatory markers showed significantly higher expression levels in the argon group at 24 h post interventionem. The cytokines IL-1β and IL-6 are regularly secreted by neurons and glia in response to an ischemic stimulus and are involved in macrophage attraction and binding [20]. Especially IL-1β is actively produced and released in stroke in tattered neurons and activated microglia by activation of inflammasomes and apoptotic pathways in the hypoxic brain. These differences in gene expression seem contradictory to the neuroprotective effects exerted by argon in tMCAO in rats [10]. The cytokines have mostly been found to aggravate tissue damage in ischemia [20-22]. IL-6 has also been attributed a protective role [23,24]. About both the underlying pathomechanisms and the prospects of these unexpected results we may only speculate. Direct as well as subordinate effects of argon on the injured tissue might be underlying these findings. Of note, expression kinetics of these cytokines showed an immediate up-regulation approximately one to two hours after brain injury induction with peak levels after 6 to 12 h [25,26]. Since in our study mRNA levels were measured with a delay of 24 h after reperfusion, it might be thinkable that besides a prolonged upregulation of these cytokines, argon might induce a delay of mRNA-expression which might effect a shift of peak expression to a later time point. At 24 h post-ischemia the elevated expression of iNOS, whose role in ischemic brain damage and repair is still discussed [27,28], might also be due to altered cytokine kinetics. Another important point to consider is that the timing of sampling might contribute to these confusing results. Only having the 24-hour snap shot might not be sufficient to draw mechanistic conclusions. Both earlier (first 6-to-12 hours of post-ischemia) and later (48 hours post-ischemia) sampling will have to be evaluated in further studies to examine these seemingly controversial results and their link to tissue protection.

Growth factors are involved in initiation of repair mechanisms to organize tissue damage and initiate cell migration and differentiation. TGF-β-expression was found to be elevated 24 h after tMCAO + argon while it was not affected by tMCAO + placebo. TGF-β is associated with profound neuroprotective activities [29,30]. NGF, which was also induced by argon treatment in our study, has been shown to exert neuroprotective properties partly via an antiapoptotic mechanism [31]. VEGFα plays a role in regulation of cerebral blood flow and angiogenesis and thereby is proposed to act as a neuroprotectant [32]. The fact that these neuroprotective factors were increased following argon therapy might contribute to its beneficial effects [10].

Tissue protection by xenon preconditioning and treatment has been associated with an up-regulation of HIF-1α in several *in vivo* and *in vitro* models of ischemia [18,33,34]. Argon preconditioning did not induce HIF-1α in renal ischemia *in vitro*, but also lacked protective effects in this experiment [18]. In our study, we did not find an upregulated expression of HIF-1α following argon treatment of tMCAO induced cerebral ischemia despite of it’s neuroprotective effects [10]. Whether the missing induction of HIF-1α is specific to argon, or due to other reasons (timing of treatment, examination time) will have to be determined elsewhere.

Still, some open questions remain: There was no statistically significant difference in neuron numbers between tMCAO + argon and tMCAO + placebo in the penumbra, although behavioural scoring and cortical infarct volumes in tMCAO + argon animals had been improved compared to placebo treatment [10]. A higher number of animals might be required to establish such a correlation. Alternatively, the reason for this finding could be that the behavioural scoring does not reflect the neuronal situation in the penumbra. Furthermore, we found several notable changes in the expression of the above named genes, but their exact cellular source remains to be identified. Additional studies will have to address these questions.

## Conclusion

In our study, we found controversial results. Besides elevated expressions of several inflammatory cytokines (IL-1β, IL-6, iNOS) we found an increased expression of neuroprotective growth factors (TGFβ, NGF, VEGF) following tMCAO + argon compared to tMCAO + placebo. These findings might be associated with the previously described neuroprotective effects of argon following experimental neurotrauma. Further studies will have to evaluate the relevance of these findings.

## Competing interests

MC and RR received lecture and consultant fees from Air Liquide Santé International, a company interested in developing clinical applications for medical gases, including argon and xenon. AVF received a travel recompensation for an investigators meeting from Air Liquide Santé International. All other authors declare that they have no competing interests.

## Authors’ contributions

YMR conducted the animal experiments. AVF, AP and NG conducted the laboratory experimental work. JG and HH performed the histological evaluation and helped to draw the manuscript. YMR and AVF performed the statistical analysis and drafted the manuscript. RR, MC, and CB participated in the study design and coordination and helped to draft the manuscript. All authors read and approved the final manuscript.

## References

[B1] Jauch EC Saver JL Adams HP Jr Bruno A Connors JJ Demaerschalk BM Khatri P McMullan PW Jr Qureshi AI Rosenfield K Scott PA Summers DR Wang DZ Wintermark M Yonas H American Heart Association Stroke Council; Council on Cardiovascular Nursing; Council on Peripheral Vascular Disease; Council on Clinical Cardiology Guidelines for the early management of patients with acute ischemic stroke: a guideline for healthcare professionals from the American Heart Association/American Stroke Association Stroke 2013 44 870-947 10.1161/STR.0b013e318284056a 23370205

[B2] The European Stroke Organisation (ESO) Executive Committee and the ESO Writing Committee Guidelines for Management of Ischaemic Stroke and Transient Ischaemic Attack 2008 Cerebrovasc Dis 2008 25 457 507 18477843 10.1159/000131083

[B3] Bandera E Botteri M Minelli C Sutton A Abrams KR Latronico N Cerebral blood flow threshold of ischemic penumbra and infarct core in acute ischemic stroke: a systematic review Stroke 2006 37 1334 1339 10.1161/01.STR.0000217418.29609.22 16574919

[B4] Dickinson R Franks NP Bench-to-bedside review: Molecular pharmacology and clinical use of inert gases in anesthesia and neuroprotection Crit Care 2010 14 229 10.1186/cc9051 20836899 PMC2945072

[B5] Sheng SP Lei B James ML Lascola CD Venkatraman TN Jung JY Maze M Franks NP Pearlstein RD Sheng H Warner DS Xenon neuroprotection in experimental stroke: interactions with hypothermia and intracerebral hemorrhage Anesthesiology 2012 117 1262 1275 10.1097/ALN.0b013e3182746b81 23143806

[B6] Brücken A Cizen A Fera C Meinhardt A Weis J Nolte K Rossaint R Pufe T Marx G Fries M Argon reduces neurohistopathological damage and preserves functional recovery after cardiac arrest in rats Br J Anaesth 2013 110 Suppl 1 i106 i112 10.1093/bja/aes509 23393152

[B7] Zhuang L Yang T Zhao H Fidalgo AR Vizcaychipi MP Sanders RD Yu B Takata M Johnson MR Ma D The protective profile of argon, helium, and xenon in a model of neonatal asphyxia in rats Crit Care Med 2012 40 1724 1730 10.1097/CCM.0b013e3182452164 22610177

[B8] David HN Haelewyn B Degoulet M Colomb DG Jr Risso JJ Abraini JH Ex vivo and in vivo neuroprotection induced by argon when given after an excitotoxic or ischemic insult PLoS One 2012 7 e30934 10.1371/journal.pone.0030934 22383981 PMC3285153

[B9] Harris K Armstrong SP Campos-Pires R Kiru L Franks NP Dickinson R Neuroprotection against traumatic brain injury by xenon, but not argon, is mediated by inhibition at the N-methyl-D-aspartate receptor glycine site Anesthesiology 2013 119 1137 1148 10.1097/ALN.0b013e3182a2a265 23867231

[B10] Ryang YM Fahlenkamp AV Rossaint R Wesp D Loetscher PD Beyer C Coburn M Neuroprotective effects of argon in an in vivo model of transient middle cerebral artery occlusion in rats Crit Care Med 2011 39 1448 1453 10.1097/CCM.0b013e31821209be 21336110

[B11] Behnke AR Yarbrough OD Respiratory resistance, oil-water solubility, and mental effects of argon, compared with helium and nitrogen Am J Physiol 1939 126 409 415

[B12] Fahlenkamp AV Rossaint R Haase H Al Kassam H Ryang YM Beyer C Coburn M The noble gas argon modifies extracellular signal-regulated kinase 1/2 signaling in neurons and glial cells Eur J Pharmacol 2012 674 104 111 10.1016/j.ejphar.2011.10.045 22094065

[B13] Koizumi J Yoshida Y Nakazawa T Ooneda G Experimental studies of ischemic brain edema: I: A new experimental model of cerebral embolism in which recirculation can be introduced into the ischemic area Jpn J Stroke 1986 8 1 8 10.3995/jstroke.8.1

[B14] Bederson JB Pitts LH Tsuji M Nishimura MC Davis RL Bartkowski H Rat middle cerebral artery occlusion: evaluation of the model and development of a neurologic examination Stroke 1986 17 472 476 10.1161/01.STR.17.3.472 3715945

[B15] Bederson JB Pitts LH Germano SM Nishimura MC Davis RL Bartkowski HM Evaluation of 2,3,5-triphenyltetrazolium chloride as a stain for detection and quantification of experimental cerebral infarction in rats Stroke 1986 17 1304 1308 10.1161/01.STR.17.6.1304 2433817

[B16] Kramer M Dang J Baertling F Denecke B Clarner T Kirsch C Beyer C Kipp M TTC staining of damaged brain areas after MCA occlusion in the rat does not constrict quantitative gene and protein analyses J Neurosci Methods 2010 187 84 89 10.1016/j.jneumeth.2009.12.020 20064557

[B17] Swanson RA Morton MT Tsao-Wu G Savalos RA Davidson C Sharp FR A semiautomated method for measuring brain infarct volume J Cereb Blood Flow Metab 1990 10 290 293 10.1038/jcbfm.1990.47 1689322

[B18] Rizvi M Jawad N Li Y Vizcaychipi MP Maze M Ma D Effect of noble gases on oxygen and glucose deprived injury in human tubular kidney cells Exp Biol Med (Maywood) 2010 235 886 891 10.1258/ebm.2010.009366 20472713

[B19] Loetscher PD Rossaint J Rossaint R Weis J Fries M Fahlenkamp A Ryang YM Grottke O Coburn M Argon: neuroprotection in in vitro models of cerebral ischemia and traumatic brain injury Crit Care 2009 13 R206 10.1186/cc8214 20017934 PMC2811924

[B20] Huang J Upadhyay UM Tamargo RJ Inflammation in stroke and focal cerebral ischemia Surg Neurol 2006 66 232 245 10.1016/j.surneu.2005.12.028 16935624

[B21] Denes A Pinteaux E Rothwell NJ Allan SM Interleukin-1 and stroke: biomarker, harbinger of damage, and therapeutic target Cerebrovasc Dis 2011 32 517 527 10.1159/000332205 22104408

[B22] Tarkowski E Rosengren L Blomstrand C Wikkelso C Jensen C Ekholm S Tarkowski A Early intrathecal production of interleukin-6 predicts the size of brain lesion in stroke Stroke 1995 26 1393 1398 10.1161/01.STR.26.8.1393 7631343

[B23] Tilg H Trehu E Atkins MB Dinarello CA Mier JW Interleukin-6 (IL-6) as an anti-inflammatory cytokine: induction of circulating IL-1 receptor antagonist and soluble tumor necrosis factor receptor p55 Blood 1994 83 113 118 8274730

[B24] Ali C Nicole O Docagne F Lesne S MacKenzie ET Nouvelot A Buisson A Vivien D Ischemia-induced interleukin-6 as a potential endogenous neuroprotective cytokine against NMDA receptor-mediated excitotoxicity in the brain J Cereb Blood Flow Metab 2000 20 956 966 10894179 10.1097/00004647-200006000-00008

[B25] Wang X Yue TL Young PR Barone FC Feuerstein GZ Expression of interleukin-6, c-fos, and zif268 mRNAs in rat ischemic cortex J Cereb Blood Flow Metab 1995 15 166 171 10.1038/jcbfm.1995.18 7798334

[B26] Wang X Yue TL Barone FC White RF Gagnon RC Feuerstein GZ Concomitant cortical expression of TNF-alpha and IL-1 beta mRNAs follows early response gene expression in transient focal ischemia Mol Chem Neuropathol 1994 23 103 114 10.1007/BF02815404 7702701

[B27] Prüss H Prass K Ghaeni L Milosevic M Muselmann C Freyer D Royl G Reuter U Baeva N Dirnagl U Meisel A Priller J Inducible nitric oxide synthase does not mediate brain damage after transient focal cerebral ischemia in mice J Cereb Blood Flow Metab 2008 28 526 539 10.1038/sj.jcbfm.9600550 17851454

[B28] Kidd GA Hong H Majid A Kaufman DI Chen AF Inhibition of brain GTP cyclohydrolase I and tetrahydrobiopterin attenuates cerebral infarction via reducing inducible NO synthase and peroxynitrite in ischemic stroke Stroke 2005 36 2705 2711 10.1161/01.STR.0000190000.98707.6d 16282548

[B29] Ruocco A Nicole O Docagne F Ali C Chazalviel L Komesli S Yablonsky F Roussel S MacKenzie ET Vivien D Hbuissob A A transforming growth factor-beta antagonist unmasks the neuroprotective role of this endogenous cytokine in excitoxic and ischemic brain injury J Cereb Blood Flow Metab 1999 19 1345 1353 10598939 10.1097/00004647-199912000-00008

[B30] Dhandapani KM Brann DW Transforming growth factor-beta: a neuroprotective factor in cerebral ischemia Cell Biochem Biophys 2003 39 13 22 10.1385/CBB:39:1:13 12835526

[B31] Yang JP Liu HJ Yang H Feng PY Therapeutic time window for the neuroprotective effects of NGF when administered after focal cerebral ischemia Neurol Sci 2011 32 433 441 10.1007/s10072-011-0512-9 21409508

[B32] Yang J Guo L Liu R Liu H Neuroprotective effects of VEGF administration after focal cerebral ischemia/reperfusion: dose response and time window Neurochem Int 2012 60 592 596 10.1016/j.neuint.2012.02.020 22394692

[B33] Limatola V Ward P Cattano D Gu J Giunta F Maze M Ma D Xenon preconditioning confers neuroprotection regardless of gender in a mouse model of transient middle cerebral artery occlusion Neuroscience 2010 165 874 881 10.1016/j.neuroscience.2009.10.063 19895874

[B34] Zhao H Watts HR Chong M Huang H Tralau-Stewart C Maxwell PH Maze M George AJ Ma D Xenon treatment protects against cold ischemia associated delayed graft function and prolongs graft survival in rats Am J Transplant 2013 13 2006 2018 10.1111/ajt.12293 23710625 PMC3884761

